# Effect of Waters Enriched in O_2_ by Injection or Electrolysis on Performance and the Cardiopulmonary and Acid–Base Response to High Intensity Exercise

**DOI:** 10.3390/nu13124320

**Published:** 2021-11-29

**Authors:** Frédéric N. Daussin, François Péronnet, Antoine Charton, Evelyne Lonsdorfer, Stéphane Doutreleau, Bernard Geny, Ruddy Richard

**Affiliations:** 1 ULR 7369–URePSSS—Unité de Recherche Pluridisciplinaire Sport Santé Société, Univ. Lille, Univ. Artois, Univ. Littoral Côte d’Opale, F-59000 Lille, France; 2 École de Kinésiologie et des Sciences de L’activité Physique, Faculté de Médecine, Université de Montréal, Montréal, QC H3C 3J7, Canada; francois.peronnet@umontreal.ca; 3 Department of Anaesthesia and Critical Care, EA 3072, University of Strasbourg, Hautepierre University Hospital, F-67091 Strasbourg, France; acharton@hotmail.fr; 4 Physiology Department, EA 3072, Faculty of Medicine, University of Strasbourg, F-67091 Strasbourg, France; Evelyne.lonsdorfer@chru-strasbourg.fr (E.L.); bernard.geny@chru-strasbourg.fr (B.G.); 5 Department of Physiology and Functional Explorations, CHRU Strasbourg, F-67091 Strasbourg, France; 6 Department of Physiology, INSERM U1042, F-38000 Grenoble, France; sdoutreleau@chu-grenoble.fr; 7 CRNH Auvergne, INRA UMR1019 and Department of Sport Medicine and Functional Explorations, CHU Clermont-Ferrand, F-63003 Clermont-Ferrand, France; ruddy.richard@uca.fr

**Keywords:** performance, reactive oxygen species, VO_2_ kinetics, endurance exercise

## Abstract

Several brands of water enriched with O_2_ (O_2_-waters) are commercially available and are advertised as wellness and fitness waters with claims of physiological and psychological benefits, including improvement in exercise performance. However, these claims are based, at best, on anecdotal evidence or on a limited number of unreliable studies. The purpose of this double-blind randomized study was to compare the effect of two O_2_-waters (~110 mg O_2_·L^−1^) and a placebo (10 mg O_2_·L^−1^, i.e., close to the value at sea level, 9–12 mg O_2_·L^−1^) on the cardiopulmonary responses and on performance during high-intensity exercise. One of the two O_2_-waters and the placebo were prepared by injection of O_2_. The other O_2_-water was enriched by an electrolytic process. Twenty male subjects were randomly allocated to drink one of the three waters in a crossover study (2 L·day^−1^ × 2 days and 15 mL·kg^−1^ 90 min before exercise). During each exercise trial, the subjects exercised at 95.9 ± 4.7% of maximal workload to volitional fatigue. Exercise time to exhaustion and the cardiopulmonary responses, arterial lactate concentration and pH were measured. Oxidative damage to proteins, lipids and DNA in blood was assessed at rest before exercise. Time to exhaustion (one-way ANOVA) and the responses to exercise (two-way ANOVA [Time; Waters] with repeated measurements) were not significantly different among the three waters. There was only a trend (*p* = 0.060) for a reduction in the time constant of the rapid component of VO_2_ kinetics with the water enriched in O_2_ by electrolysis. No difference in oxidative damage in blood was observed between the three waters. These results suggest that O_2_-water does not speed up cardiopulmonary response to exercise, does not increase performance and does not trigger oxidative stress measured at rest.

## 1. Introduction

At equilibrium with partial pressure at sea level (PO_2_ ~160 mmHg) and for temperature ranging between 10 and 25 °C, the amount of O_2_ dissolved in water ranges respectively between 12 and 9 mg·L^−1^ [1] (Box 1). However, several commercially available brands of water are purportedly enriched in O_2_ (O_2_-waters) at 7 and 40 times this basal value [2,3,4] and are advertised as wellness and fitness waters with a large range of physiological and psychological benefits. As discussed by several authors [2,3,5,6], these claims are based at best on anecdotal evidences or on a limited number of unreliable studies.

Box 1 **Relationship between PO_2_ and temperature, and O_2_ content of water according to Henry’s law [1] expressed in various units: mol·L^−1^, mL·L^−1^, mg·L^−1^ and ppm, at sea level, 10 °C and dry air.**

The amount of O_2_ dissolved in water (mol·L^−1^) depends on the temperature (T in K = T[°C] + 273) and PO_2_ (in atmosphere: atm) and is described by Henry’s law where 769.23 and 1700 are Henry’s constants for O_2_:

   O_2_ content (mol·L^−1^) = PO_2_ (atm)/{769.23 × exp [−1700 × (1/T − 1/298)}

For example, at sea level (PO_2_ = 160 mmHg or 0.21 atm in dry air) and 10 °C (283 K):

   O_2_ content (mol.L^−1^) = 0.21 (atm)/{769.23 × exp [−1700 × (1/283 − 1/298)} = 0.000370 mol·L^−1^

Since the molar mass of O_2_ is 32 g and since 1 mol of O_2_ occupies 22.4 L, the conversion of this O_2_ content in various other units is as follows:

   O_2_ content in mL·L^−1^ = O_2_ content in mol·L^−1^ × 22.4 × 1000 = 0.000370 × 22.4 × 1000 = 8.29 mL·L^−1^

   O_2_ content in mg·L^−1^ = O_2_ content in mol·L^−1^ × 32/1000 = 0.000370 × 32/1000 = 11.8 mg·L^−1^

Finally, since the molar mass of water is 18 g, there are 1000/18 = 55.5 mol of water·L^−1^. The O_2_ content in ppm, thus is:

   O_2_ content in ppm = O_2_ content in mol·L^−1^/H_2_O content in mol·L^−1^ = (0.000370/55.5) × 10^6^ = 6.67 ppm

Consumption of O_2_-waters is promoted in active subjects, and a dozen studies have been conducted to document their effects, if any, on the response to exercise and on performance (Table 1). However, in some of these studies, the characteristics of the water ingested, including the O_2_-content, were not precisely described, and the volume ingested was not indicated. In only two studies [2,7], the O_2_ content measured and/or reported appears reliable and in the range of values for which benefits are claimed by the manufacturers. In these two studies, O_2_-waters consumption did not improve maximal oxygen consumption (VO_2_max) [2,7] and in the study by Leibetseder et al. [7], the only significant difference when the O_2_-water was ingested was an increase in the ventilatory equivalent of oxygen (pulmonary ventilation/O_2_ consumption or VE/VO_2_) at sub-maximal exercise and in plasma lactate concentration at maximal exercise. As discussed by the authors, it is difficult to speculate about the physiological significance of these changes, their possible effects on performance and how they could be related to ingestion of the O_2_-water, but these findings suggest that O_2_-waters could modify the cardiopulmonary response to exercise.

As already discussed [2,4,8], ingestion of water enriched in O_2_ is unlikely to directly modify oxygen consumption (VO_2_) at the mouth during exercise. In addition, in arterial blood, the amount of dissolved O_2_ is very small compared to the amount carried by hemoglobin, and in healthy subjects exercising at sea-level or a low altitude (<1000 m), arterial hemoglobin saturation remains close to normal values. It is, thus, very unlikely that increasing O_2_ delivery to the blood through the gut will markedly increase PO_2_ and O_2_ content in arterial blood and, thus, O_2_ delivery to any organ or tissue, including exercising muscles. However, we have previously studied the effects of a water enriched in O_2_, using an electrolytic process [9,10,11]. When compared to injection of O_2_, this process could generate water superstructures called clathrates, which could trap solutes, such as O_2_, and which can facilitate O_2_ diffusion along PO_2_ gradients [12,13,14]. This hypothesis is consistent with the higher tissue oxygenation observed in anaesthetized pigs [11] and the higher mitochondrial respiration observed at low PO_2_ in permeabilized rat muscle fibers [10] with water enriched by electrolysis vs. injection.

It have shown that the fast component of VO_2_ kinetics at the mouth in response to constant load exercise reflects O_2_ diffusion from the blood to muscle fibers [15]. Therefore, the aim of this study was to investigate the effect of O_2_-waters on the cardiopulmonary and acid–base responses during near maximal exercise continued to exhaustion. We hypothesized that the fast component of the kinetics of VO_2_ in response to high-intensity exercise could be faster following ingestion of the water enriched in O_2_ by electrolysis vs. injection or a control water with a low O_2_ content. A faster adjustment of VO_2_ at the onset of exercise could increase the time to exhaustion. Finally, it has been suggested that ingestion of O_2_-waters can be a safety issue because of production of reactive oxygen species (ROS) [5,6,16,17]. We, thus, also investigated the presence of ROS induces damages, including DNA damages, using the comet assay.

## 2. Methods

### 2.1. Subjects

Twenty active and healthy male subjects, recruited through advertisements in the hospital and university communities, volunteered for this study, which was approved by the local ethic committee (Comité de Protection des Personnes Est IV, Strasbourg, France: Eudract No.2008-A01051-54). Their age, height, body mass and percent body fat were respectively 22.8 ± 4.1 years, 178.7 ± 5.9 cm, 70.0 ± 5.7 kg and 13.0 ± 1.6% (mean ± SD). In accordance with the declaration of Helsinki, all participants signed an informed-consent form. The sample size was determined based on the significant difference in plasma lactate concentration at maximal exercise between the control and O_2_-water by Leibetseder et al. [7] (9.6 vs. 11.1 mmol·L^−1^ with SD = 1.6 mmol·L^−1^). These data indicated that 18 subjects were needed to detect a difference with an 80% power and *p* = 0.05 [25].

### 2.2. Study Design

The participants visited the laboratory on four separate occasions. All experimental conditions were conducted in a climate-controlled laboratory at Strasbourg Hospital. The first session was used to determine the VO_2_max and maximal power output by an incremental test to exhaustion. On the subsequent three visits separated by at least 5 days, the subjects performed an exercise trial to volitional fatigue. The study was randomized and double blind. The cardiopulmonary and acid–base response and time to exhaustion were measured.

### 2.3. Determination of VO_2_max and Pmax

VO_2_max and the corresponding workload (Pmax) were measured before the first experimental trial on cycle ergometer (Ergoline 900, Ergoline, Schiller, France), using an incremental test to volitional fatigue, as previously described [26]. The VO_2_ and carbon dioxide production (VCO_2_) were computed from breath-by-breath measurement of gas exchanges at the mouth (Sensor Medics, Yorba Linda, CA, USA) and the heart rate was measured from a 12-lead ECG (Cardiovit CS200, Schiller, Baar, Switzerland). The Pmax was considered to be the lowest workload, eliciting VO_2_max = 3.63 ± 0.39 L O_2_·min^−1^, reached at 276 ± 31 W, with heart rate (HR) = 187 ± 7 bpm and plasma lactate concentration = 12.4 ± 3.3 mmol·L^−1^.

### 2.4. O_2_-Waters Ingested

The waters ingested were prepared from demineralized water, which was remineralized with Na^+^, SO_4_^2−^ and PO_4_^2−^. The control water was enriched in O_2_ by injection (10 mg·L^−1^, i.e., close to the value at equilibrium with atmospheric O_2_ at sea level at the temperature of ingestion (5–10 °C)). The two other waters were enriched at ~110 mg·L^−1^ by injection or electrolysis, as previously described [11]. Their O_2_ content was found to be 116 and 109 mg·L^−1^, respectively, for the water enriched in O_2_ by electrolysis and injection. A very good stability of O_2_ was found in the two O_2_-waters. Upon opening and keeping the bottle unagitated at 20 °C, the half-life of the decrease in O_2_ content was about six days.

### 2.5. Exercises with Control and O_2_-Waters

The subjects took part in three similar exercise trials by 5- to 7-day intervals. For two days before each trial, the subjects ingested 2 L·day^−1^ of the assigned water (~500 mL every 4 h during the wake-up period). The waters were kept in a refrigerator (~5 °C) and were consumed within ~15 min, following removal of the cap. The subjects also ingested 15 mL·kg^−1^ of the same water 90 min before the beginning of the exercise trial. Over the two-day period preceding each exercise trial, the subjects refrained from exercising and were fed a standardized diet (35 kcal·kg^−1^·day^−1^ with 15, 35 and 50% energy from protein, fat and carbohydrate).

During each exercise trial, following a 20-min warm-up and a 5-min rest period, the subjects exercised at Pmax to volitional fatigue. Breath-by-breath gas exchanges were measured for the computation of ventilation (VE), breathing frequency (fR), tidal volume (VT), VO_2_, VCO_2_/VO_2_ (RER) and VE/VO_2_. The HR was monitored from a 3-lead ECG (Nihon Kohden TEC-5500, Tokyo, Japan), and cardiac output (Qc) was continuously monitored by impedancemetry (Physioflow, Manatec, France). The stroke volume and arterio-venous difference in O_2_ were computed. Finally, 100 µL blood samples were withdrawn from the arterialized earlobe at rest before exercise and every minute during the exercise period for the measurement of plasma lactate concentration and pH, arterial oxygen and carbon dioxide pressure (PaO_2_ and PaCO_2_) and arterial oxygen saturation (SaO_2_) (Bayer, Bayer series 800, Bayer France; Instrumentation Laboratory, Company, Bedford, MA, USA). PO_2_ measured in blood sampled at the arterialized earlobe was corrected, as previously suggested [27] (PaO_2_ [in mmHg] = 1.1 × earlobe PO_2_ − 3).

### 2.6. VO_2_ Kinetics

The time course of the VO_2_ response at the onset of exercise was described by using a three-component model [28]. Due to methodological considerations, the cardiodynamic phase was excluded by removing the data points in the first 20 s from the analysis [29]. We used a mathematical model with two exponential functions [30]. The parameters of the model were determined using an iterative procedure which minimizes the sum of the mean squares of the differences between VO_2_ estimates and the corresponding actual values as previously described [31]. The amplitude of the slow component was computed as suggested by Borrani et al. [32]. Aberrant breaths were excluded from the analysis, as previously described [33]. Less than 1% of the data points were excluded.

### 2.7. ROS Generation and Damages

Blood samples (6 mL) were taken at rest before exercise in an antecubital vein, following ingestion of the waters. These samples were assessed for estimating oxidative stress on lipid species (blood malondialdehyde (MDA) concentration [34]), protein species (preserved thiol groups [34]) and on endogenous antioxidant defenses (reduced and oxidized glutathione [35]; and on DNA [36]).

### 2.8. Statistical Analyses

Data are reported as mean ± standard deviation (SD). The normality of distribution was verified by using the Shapiro–Wilk test. Comparisons were made by using ANOVA for repeated measurements (one- or two-way [Time; Water], depending on the variable; see results) and Tukey HSD at *p* < 0.05 (SAS Software version 9.1, SAS Institute, Cary, NC, USA). The effect of time was tested by comparing the values observed before exercise to those observed at min 5:00 and the end of the exercise period, except for RER, for which the comparison was made between the final value and the peak value observed at min 3:30. A two-sided level of 5% for the type 1 error was applied. A significance level between 5 and 10% was considered as a trend.

## 3. Results

**Figure 1:**
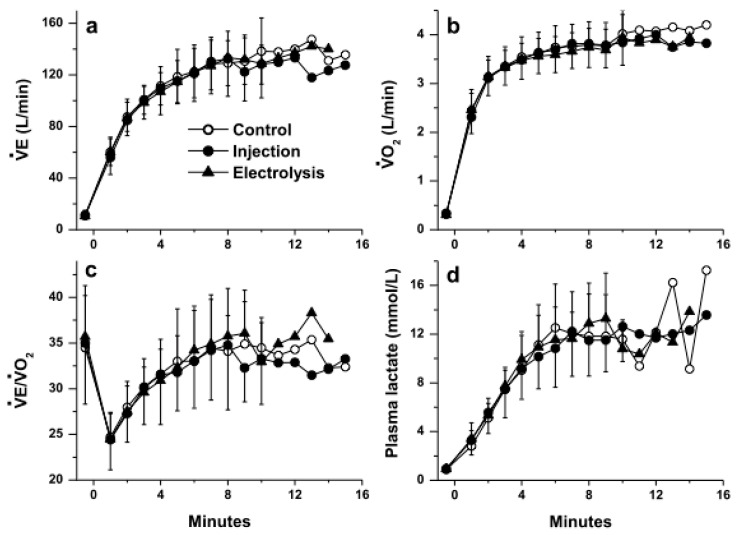
Pulmonary responses during exercise: (**a**) pulmonary ventilation (VE), (**b**) oxygen uptake (VO_2_), (**c**) ventilatory equivalent of oxygen and (**d**) plasma lactate concentration with the three waters (mean ± SD; SD not shown past min 10 because n < 5; no significant difference was observed between the three waters, *p* > 0.05).

**Figure 2:**
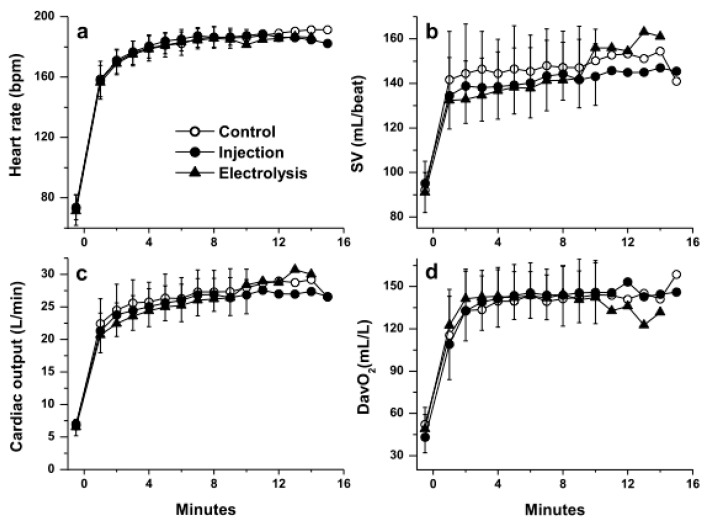
Circulatory responses during exercise: (**a**) heart rate, (**b**) stroke volume (SV), (**c**) cardiac output and (**d**) arterio-venous difference in O_2_ (DavO_2_) with the three waters (mean ± SD; SD not shown past min 10 because *n* < 5; no significant difference was observed between the three waters, *p* > 0.05).

Figure 1 and Figure 2 show the kinetics of VE, VO_2_, VE/VO_2_, lactate concentration and of the circulatory adjustments in response to exercise, respectively. Table 2 shows the values of PaO_2_, PaCO_2_, SaO_2_ and pH observed at rest before exercise, at min 5 during the exercise and at the end of exercise.

As expected, in response to high-intensity exercise continued to volitional fatigue, following an early response observed between min 0 and min ~5, some variables levelled off (VT [data not shown], VE/VO_2_, plasma lactate concentration, pH and PaO_2_), while others significantly drifted upwards (fR and RER [data not shown], and VCO_2_, VE, VO_2_, HR and Qc) or downwards (PaCO_2_ and SaO_2_) (Figure 1 and Figure 2). No significant difference was observed between the three waters for any of the variables measures at any time points.

No significant difference was observed for any of the parameters of the VO_2_ kinetics between the three waters. However, there was a trend for the time constant of the fast component of the kinetics of VO_2_ (τ_1_) to be different with the three waters (*p* = 0.060) due to the large difference between the values computed with the control water and the water enriched in O_2_ by electrolysis (Tukey HSD, *p* = 0.0513; the corresponding *p*-values for the comparison between the control water and the water enriched by injection, and between the two waters enriched in O_2_ were much larger, at 0.668 and 0.272, respectively) (Table 3).

No significant difference was observed between exercise time to exhaustion with ingestion of the three waters (in min:s: 10:28 ± 3:52, 9:28 ± 3:42, and 9:54 ± 3:38 with control water and the waters enriched in O_2_ by injection and the electrolytic process), and the values in the three trials were closely correlated (injection vs. control: Pearson r = 0.825; electrolysis vs. control: Pearson r = 0.847; electrolysis vs. injection: Pearson r = 0.875; *p* < 0.001 for the three correlation coefficients).

No significant difference was observed between the three waters for any of the indices of ROS generation and damages at rest before exercise (Table 4).

## 4. Discussion

The results from the present experiment indicate that ingestion of waters enriched in O_2_ by injection or electrolysis at ~11 times the level in the control water did not modify the cardiopulmonary response to high-intensity exercise, the response of plasma lactate concentration and pH, or the response of arterial blood gases. No significant difference was observed between the three waters for the parameters of the kinetics of VO_2_; however, there was a trend for the fast component to be slightly faster with the water enriched in O_2_ by the electrolytic process. Finally, ingestion of waters enriched in O_2_ does not increase time to exhaustion at high exercise intensity, but it also has no adverse effect on the production of ROS and associated damages to lipids, proteins or DNA measured at rest.

These observations are difficult to compare with data in the literature; however, a dozen studies have described the effect of O_2_-water consumption on the response to exercise (Table 1). In only two of these studies [2,7], the O_2_ content, which was reported or was computed from the PO_2_ reported (see Box 1), was higher than the minimal value for which benefits have been claimed in terms of physiological response and performance (about >7 times the value in tap water) and were credible. In three of the ten other studies listed in Table 1, the O_2_ content of the water ingested was simply not reported [18,20,23]. In four other studies [8,17,21,24], there was no evidence that the O_2_ content was actually measured, and the values reported are much too high to be credible: the O_2_ content reported would require PO_2_ in excess of ~900 to ~10,000 atmospheres. Finally, in the three studies listed, in which the O_2_ content in commercially available O_2_-waters was actually measured [4,19,37], its value was found to be much lower than advertised by the manufacturer and similar to or only slightly above the value at equilibrium at sea level (see Box 1). It is also worth mentioning that, in only one of the five brands of O_2_-waters analyzed by Hampson et al. [2], the PO_2_ corresponded to an O_2_ content higher than ~4× the content at equilibrium at sea level. Taken together, results from 10 of the 12 studies available in the literature do not allow us to conclude about putative effects of O_2_-waters on the response to exercise and on performance, because the O_2_-content cannot be ascertained, is incredibly high, or is much too low.

As already discussed by several authors [3,4,7], when compared to the VO_2_ at rest and even more during exercise, the amount of O_2_ which could be supplied to the blood in the gut by O_2_-waters is extremely small, and unlikely to directly increase aerobic energy production at rest and even less during exercise. In addition, as observed by Fleming et al. [8], a putative increase in VO_2_ in peripheral tissues because of an additional supply of O_2_ from ingested O_2_-water will not be detected from VO_2_ measurement at the mouth which only tracks pulmonary gas exchanges. It is, thus, not surprising that, in previous studies [2,7], as well as in the present experiment, for a given workload, the VO_2_ was similar between the three waters. The only previous significant differences reported were a slightly higher VE/VO_2_ at submaximal workload and a higher plasma lactate concentration at maximal exercise [7]. However, the differences were low, and the authors questioned their physiological significance. In the present experiment, O_2_-waters with an O_2_ content well above the range of values for which manufacturers claim benefits in terms of physiological response and performance did not have any effect on any of the variables measured. Taken together, these data question the interest to use O_2_-water as an ergogenic aid to improve aerobic performance.

There was, however, a trend for the water enriched in O_2_ by electrolysis to speed up the kinetic of VO_2_ in response to high-intensity exercise when compared with the control water. Since the fast component of the kinetics of VO_2_ reflects O_2_ diffusion from the blood to muscle fibers [15], this observation is consistent with our previous study showing a lower apparent Km of the mitochondria for O_2_ in a solution enriched in O_2_ by electrolysis, without any change in the Km for ADP in rat permeabilized muscle fibers [10]. The faster diffusion of O_2_ to the mitochondria, as well as the trend to faster adjustment of VO_2_ at the onset of exercise observed in the present experiment, in turn, is consistent with the hypothesis that the electrolytic process could generate supramolecular water structures, similar to clathrates [12,13,14], which can trap O_2_ molecules and could modify the local pressure/content relationship for O_2_.

Finally, it has been suggested that administering O_2_ internally by ingesting O_2_-waters, which increases PO_2_ in the portal vein in rats [38], can be a safety issue because of possible damage to the liver and generation of oxygen radicals [16]. Previous studies showed that acute ingestion of O_2_-waters (300 mL with O_2_ content ranging from 30 to ~200 mg·L^−1^) transiently increased blood ascorbyl radical concentration [5,6]. This effect was attenuated following chronic ingestion (0.9 to 1.5 L·day^−1^ for 21 to 28 days). Moreover, ingestion of an O_2_-water for eight days significantly increased serum lipid peroxide concentration and urine alkenyl excretion [17]. However, as already discussed, the actual O_2_ content in the water ingested in this study cannot be ascertained. In the present experiment, ingestion of O_2_-water did not result in ROS-induced damage at rest. In addition, the comet assay, which is very sensitive to detect oxidative DNA damages, did not show any genotoxicity of the O_2_-waters administered. A similar result was reported 30 and 60 min following ingestion of 500 mL of O_2_-waters with 70 mg O_2_·L^−1^ [39]. Taken together, these results suggest that consumption of waters enriched in O_2_ does not induce oxidative stress at rest in healthy subjects.

## 5. Conclusions

In response to high-intensity exercise, results from the present study did not show any beneficial effects of water enriched in O_2_ by injection or electrolysis on exercise time to exhaustion, on the cardiopulmonary response and on arterial lactate concentration and pH.

## Figures and Tables

**Table 1:** Studies of the effects of O_2_-water ingestion on performance and on the response to exercise.

Reference	Water Ingested	Reported (1) O_2_ Content or PO_2_	Computed (2) O_2_ Content (mg·L^−1^) and PO_2_ at 10 °C	Observations
Askew et al. 2001 [17]	Stabilized O_2_ in water Ingestion for 8 days Volume not reported	30,000 ppm	~53,700 mg·L^−1^ ~725,000 mmHg ~950 atm	No significant difference between the O_2_-water and the placebo in response to a graded exercise to VO_2_max
Duncan 1997 [18]	Oxygen-enhanced water Volume not reported	Not reported	?	5 km run (min:s) Placebo: 21:18 O_2_-water: 20:47 (not significant)
Fleming et al. 2017 [8]	Activate Stabilized Water (ASO): 0.9 L during and after exercise	35 g in 62 g of water	565 mg·L^−1^ ~7,600,000 mmHg ~10,000 atm	5-km run: Lactate clearance (t_1/2_ in seconds) Placebo: 1223 O_2_-water: 1127 (*p* < 0.05)
Fuller 2010 [19]	Activate Stabilized Water (ASO): 0.5 L during and after exercise	5 mg·L^−1^	5 mg·L^−1^ 68 mmHg 0.09 atm	Trend for a longer time to exhaustion during a graded test to VO_2_max with the O_2_-water than the placebo (451 vs. 429 s)
Hampson et al. 2003 [2]	Oxygenated water 355 mL 5 min before exercise	1,184 mmHg (3) 226 mL·L^−1^	88 mg·L^−1^ (3) 61.4 mL·L^−1^ 1.56 atm	No significant difference between the O_2_-water and the placebo in response to a graded exercise to VO_2_max
Jenkins et al. 2001 [20]	Oxygenized water 0.45 L 10 min before and after exercise	Not reported	?	Higher hemoglobin saturation in arterial blood at the end of exercise at 100%VO_2_max with the O_2_-water than the placebo (94 vs. 87%)
Leibetseder et al. 2006 [7]	Oxygenated water 1.5 L·day^−1^ for 2 weeks	160 mg·L^−1^	160 mg·L^−1^ 2150 mmHg 2.83 atm	Higher VE/VO_2_ at submaximal workload and higher lactate concentration at maximal workload with the O_2_-water than the placebo
McNaughton et al. 2007 [21]	Superoxygenated water (Oxyshot) 15 mL 30 min before exercise	150,000 ppm (4)	266,000 mg·L^−1^ ~ 3,600,000 mmHg ~ 4750 atm	No significant difference for a 45-min exercise at 70%VO_2_max followed by a 15-min time trial to exhaustion between the O_2_-water and the placebo
Mielke et al. 2004 [22]	Oxygenated water 1.2 L·day^−1^ for 3 days and 0.6 L 15 min before exercise	13.1 mg·L^−1^	13.1 mg·L^−1^ 177 mmHg 0.23 atm	No significant difference in response to a graded exercise to VO_2_max or in exercise time to exhaustion at 90%VO_2_max between the O_2_-water and the placebo
Willmert et al. 2002 [4]	Super oxygenated water 0.5 L 15 min before exercise	13.5 mL·L^−1^	19.3 mg·L^−1^ 260 mmHg 0.34 atm	No significant difference between the O_2_-water and the placebo in response to a graded exercise to VO_2_max
Wing-Gaïa et al. 2005 [23]	Purified oxygen water 35 mL·kg^−1^·day^−1^ for 3 days 0.5 L 2 h before exercise	Not reported	?	No significant difference in performance or in response to a time trial at 57–59%VO_2_max in hypoxic condition (~76 min) between the O_2_-water and the placebo
Zhang et al. 2005 [24]	Hyperoxia solution 0.25 L before exercise	170 mL·0.5 L^−1^	481 mg·L^−1^ 6500 mmHg 8.55 atm	Lower plasma lactate concentration in response to a 5 km run at altitude (2000 and 4000 m) with the O_2_-water than the placebo

(1) Values reported by the authors. (2) Values computed from the data reported by the authors using Henry’s law [1] at 10 °C. (3) The O_2_ contents computed in the study by Hampson et al. [2] from the PO_2_ measured in tap water and five brands of O_2_-waters (e.g., 226 mL·L^−1^ for 1184 mmHg in the brand 5 studied at exercise) are all in error. The correct value of O_2_-content for a PO_2_ = 1184 mmHg and at 10 °C is 61 mL·L^−1^ corresponding to 88 mg·L^−1^. The O_2_ content in «well-stirred» tap water with a PO_2_ of 127 mmHg is 6.6 mL·L^−1^ at 10 °C and 4 mL·L^−1^ at 37 °C (i.e., well below the value reported of 25 mL L^−1^) which is in line with the textbook value of 3 mL L^−1^ dissolved in arterial blood at a PO_2_ of ~100 mmHg and 37 °C [14]. (4) Not reported by the authors but found at website (https://www.reachforlife.com.au/Equine/EQ-Product.php, consulted 22 November 2021).

**Table 2:** Blood gases and pH.

	Water	Rest	Min 5	End of Exercise
PaO_2_ (mmHg)	Control	91.1 ± 8.8	86.4 ± 6.4 ^a^	86.1 ± 7.4 ^a^
	Injection	93.1 ± 9.1	84.4 ± 6.7 ^a^	84.6 ± 8.5 ^a^
	Electrolysis	94.9 ± 6.8	86.1 ± 4.7 ^a^	83.5 ± 5.0 ^a^
PaCO_2_ (mmHg)	Control	37.5 ± 1.9	30.7 ± 3.3 ^a^	27.3 ± 4.3 ^a,b^
	Injection	36.7 ± 2.2	31.0 ± 3.5 ^a^	27.6 ± 4.3 ^a,b^
	Electrolysis	37.3 ± 2.3	30.6 ± 3.1 ^a^	27.9 ± 4.0 ^a,b^
SaO_2_ (%)	Control	95.7 ± 1.2	95.0 ± 1.4	94.3 ± 1.3
	Injection	95.5 ± 1.0	94.6 ± 0.8	94.0 ± 1.5
	Electrolysis	96.1 ± 1.1	95.0 ± 0.8	93.9 ± 1.0
pH	Control	7.40 ± 0.03	7.40 ± 0.03 ^a^	7.26 ± 0.05 ^a,b^
	Injection	7.39 ± 0.04	7.30 ± 0.03 ^a^	7 26 ± 0.04 ^a,b^
	Electrolysis	7.40 ± 0.03	7.30 ± 0.03 ^a^	7.25 ± 0.04 ^a,b^

Partial pressure of O_2_ and CO_2_, hemoglobin saturation (SaO_2_) and pH in arterialized blood at rest before exercise, at min 5 during the exercise and at the end of exercise with the three waters (mean ± SD; ^a^ significantly different from rest; ^b^ significantly different from min 5, *p* < 0.05; comparisons with two-way ANOVA for repeated measurements).

**Table 3:** Pulmonary VO_2_ kinetics responses.

	Control	Injection	Electrolysis	*p*
td_1_ (s)	4.0 ± 9.8	4.9 ± 9.4	5.1 ±13.1	0.886
τ_1_ (s)	46.0 ± 15.4	43.5 ± 16.1	38.8 ± 16.8	0.060
A_1_ (mL O_2_·min^−1^)	2.98 ± 0.44	2.95 ± 0.40	2.91 ± 0.362	0.581
td_2_ (s)	208.5 ± 85.0	209.1 ± 82.3	189.9 ± 88.2	0.643
τ_2_ (s)	285.3 ± 96.0	254.6 ± 79.8	281.5 ± 101.5	0.266
A’_2_ (mL O_2_·min^−1^)	0.46 ± 0.18	0.53 ± 0.24	0.51 ± 0.20	0.773

Mean ± SD and *p*-values for the comparisons with one-way ANOVA for repeated measurements; td_1_ and td_2_, and τ_1_ and τ_2_ are respectively the time constants and the time delays for the fast and slow components of VO_2_ kinetics; A_1_ is the asymptotic amplitude of the fast component of VO_2_ kinetics; and A’_2_ is the amplitude of the slow component of VO_2_ kinetics computed as suggested by Borrani et al. [32].

**Table 4:** Indices of ROS and damages in venous blood samples taken at rest.

	Control	Injection	Electrolysis	*p*
Blood MDA content (μmol·L^−1^)	3.09 ± 0.37	3.11 ± 0.37	3.06 ± 0.43	0.682
Preserved thiol (μmol·g protein^−1^)	6.22 ± 0.34	6.24 ± 0.42	6.22 ± 0.46	0.895
Oxidized gluthatione (μmol·L^−1^)	10.1 ± 5.1	10.7 ± 8.1	11.1 ± 12.7	0.758
Reduced gluthatione (μmol·L^−1^)	933 ± 266	869 ± 137	937 ± 444	0.856
DNA damage (% tail) with FPG without FPG	4.69 ± 1.09 3.02 ± 0.74	4.77 ± 1.14 2.85 ± 0.85	4.71 ± 1.19 3.01 ± 0.70	0.600 0.140

Mean ± SD and *p*-values for the comparisons with one-way ANOVA for repeated measurements). MDA, malondialdehyde; FPG, formamidopyrimidine DNA glycosylase (DNA repair enzyme).

## Data Availability

The data that support the findings of this study are available from the corresponding author upon reasonable request.
